# Some Hospital Statistics: Their Use and Value

**Published:** 1916-08-26

**Authors:** 


### The Hospital

The Workers' Newspaper of Administrative Medicine and Institutional Life, Administration, National Insurance and Health.

No. 1577, Vol. LX. SATURDAY, AUGUST 26, 1916. PRICE ONE PENNY

### SOME HOSPITAL STATISTICS: THEIR USE AND VALUE.

It is as remarkable as it is satisfactory that during the many years Sir William Church has presided over the Distribution Committee of the Metropolitan Sunday Fund the awards made have seldom led to criticism, and the decisions of the committee have been accepted with practical unanimity. One reason for this may be that statistics do not constitute a study which commends itself to the majority of mankind. Even when a critic arises he often manifests the small amount of time and the paucity of knowledge which he has brought to bear on the subject. Most people consider they have a gift to discuss the cost per bed of this or that hospital or of hospitals generally. It is useful occasionally to point out that before an argument is based upon the cost per bed it is essential that all the factors involved, the circumstances of each hospital, the departments in which there has been an increase in the expenditure in a given period, and a multitude of other factors, have to be considered. Otherwise it is misleading and a waste of time to attempt to draw comparisons or to condemn or praise the management of a particular institution. This commonplace knowledge is unfortunately not possessed by all critics, and in the result there are not wanting those who condemn statistics giving the cost per bed, and would, if they could, discredit audited figures and the most accurate returns, though the latter may be accompanied by full explanatory notes which make them of supreme value to every intelligent hospital manager, whose first aim is efficiency, and the attainment of a full return upon all hospital money spent in each department.

King Edward's Hospital Fund for London does really good work each year by the publication of a Statistical Report on the ordinary expenditure of upwards of 100 London hospitals. This report is accompanied by groups of tables in which every type of hospital is dealt with under its proper heading. Those who are interested in the expenditure of our modern hospitals and are not familiar with these reports should make it their business to approach the Fund with a view to obtaining a complete set of them. A study of these reports, and the acquirement of a sound knowledge of how they have been built up, of the reforms which have been brought out since they were first published, and of the large reduction in expenditure which has resulted, must be impressed with the great practical value of hospital statistics when they are compiled upon an identical basis, and accompanied by explanatory notes and reports, written by experts who understand their business and have the gift of exposition. In the Statistical Report issued a year ago will be found a careful and instructive explanation of the special circumstances arising out of the war, which the Metropolitan voluntary hospitals, and, indeed, all hospitals, have had to face and provide for. The effect upon prices, especially where articles of general consumption by hospitals have usually been obtained from abroad, and others greatly in demand for the Military Medical Service, is pointed out. The bearing on the cost of hospital work of the withdrawal of many of the medical, nursing, and administrative staffs for various forms of war service, and the action of a large number of the voluntary hospitals in placing beds at the disposal of the Government for the treatment of sick and wounded sailors and soldiers, have been to create considerable changes involving increased cost all round. The latter practice has involved the keeping vacant of a number of beds set aside for naval and military patients so as to be prepared to meet any sudden influx of wounded men which war entails.

It is now generally recognised that these and other war conditions have produced variations in the cost of working a voluntary hospital which account for an increase of expenditure at one hospital and a decrease in that of another. The King's Fund authorities find that, on the whole, there has been a general increase in the cost of Metropolitan hospitals, combined with a decrease in the number of occupied beds. It follows that no statistician would attempt to compare the working of a particular hospital, or group of hospitals, in war time, with the cost of the same institutions in the years which preceded the outbreak of the Great War. Further, figures which have served in peace time as a useful measure of total changes in cost under ordinary circumstances, become, in war time, comparatively valueless, because the cost in set years may be affected by causes which are quite outside the ordinary circumstances of our voluntary hospitals. Another important consideration in dealing with the expenditure figures of our voluntary hospitals during war time is the material increase in expenditure, and in many cases of occupied beds, resulting from the admission from the Front of warrior patients.

There are, of course, isolated instances where a hospital manager, or managers, still adheres to the old doctrine that although there are grave abuses and unnecessary expenditure in certain voluntary hospitals, no such evils can or do exist, in fact, in connection with the particular hospital with which they are associated, no matter how high its average cost per bed, or its cost per bed under the main sub-headings of expenditure. Those who take up a position of this kind are usually persons who have not the faculty or the patience to go into details, much less into the elaborate details and thorough and exhaustive examination of the actual figures which the King's Hospital Fund places every year at the disposal of all hospital managers throughout the country. This may account for the few critics who seem to regard any allusion to the average cost per bed, or to sub-heads of expenditure, as impertinent, because they are so sure whatever the figures may be, that their hospital is perfect in every particular. It seems incredible that such views should be held in the present day when impartial thoroughness and a whole-hearted desire are shown to help every hospital manager by placing within his reach exhaustive and accurate figures and calculations based upon audited returns which cannot be disputed. Still, there are such people, and it is well that the Press and the public should realise this fact, for it sometimes happens that the less accurate and reliable the impressions of a particular critic may be, the more certain is it that no opportunity will be lost to try to throw discredit on statistical methods pursued in the interests of sound hospital management and the wise control of expenditure in all departments of a big hospital. The King's Hospital Fund, its Council, and officers, are entitled to the grateful recognition of the managers of every ably administered voluntary hospital. We rejoice that the overwhelming majority of these gentlemen recognise their indebtedness to the Fund for the assistance its annual Statistical Report on the ordinary expenditure of London hospitals has been, and must continue to be, to every one of them.

